# Editorial: Cerebral amyloid angiopathy: from bench to bedside

**DOI:** 10.3389/fnins.2024.1370352

**Published:** 2024-02-06

**Authors:** Hamid R. Sohrabi, Steven M. Greenberg, Luke Whiley

**Affiliations:** ^1^Centre for Healthy Ageing, Health Future Institute, Murdoch University, Perth, WA, Australia; ^2^School of Psychology, College of Health and Education, Murdoch University, Perth, WA, Australia; ^3^Massachusetts General Hospital and Harvard Medical School, Harvard University, Boston, MA, United States; ^4^Centre for Computational and Systems Medicine, Murdoch University, Perth, WA, Australia

**Keywords:** cerebral amyloid angiopathy (CAA), amyloid-related imaging abnormalities (ARIA), iatrogenic CAA (iCAA), CAA-related inflammation (CAA-ri), fluid biomarkers, peak width of skeletonized mean diffusivity (PSMD), quantitative susceptibility mapping (QSM)

Cerebral amyloid angiopathy (CAA) is a major cause of lobar intracerebral haemorrhagic stroke commonly seen in older adults (Viswanathan and Greenberg, 2011). A small percentage of CAA patients develop the autosomal dominant form of the disease due to mutations in APP (amyloid precursor protein), CST3 (Cystatin C) or ITM2B (integral membrane protein 2B) genes, among others, with relatively different presentations including haemorrhagic stroke and dementia at a younger age (Banerjee et al., 2023). Currently, CAA is understood to be caused by the gradual and progressive amyloid-beta (Aβ) deposition in the walls of small to medium-sized brain blood vessels, cerebral capillaries and leptomeningeal arteries and arterioles (Preston et al., 2003), resulting in alteration of cerebrovascular physiology, followed by non-haemorrhagic brain injury, and ending with appearance of haemorrhagic brain lesions that will result in significant impairment in cognitive abilities, change in behavior and neuropsychiatric symptoms and death (Koemans et al., 2023). Over the last 3 decades, research has paved the way to our current understanding of the CAA pathophysiological processes and its clinical presentation and diagnosis. For example, the revised Boston Criteria v2.0 represents the current state of practice proposing imaging (MRI) and clinical markers for CAA diagnosis (Charidimou et al., 2022). Also, recent findings from positron emission tomography (PET) images using amyloid tracer (e.g., C-11 Pittsburgh compound B), is a step forward to assess Aβ deposits in CAA, antemortem, and will inform monitoring of the disease and treatment efficacy, when disease modifying treatments become available (Schultz et al., 2019). Finally, research has led to a better understanding of the CAA cerebrospinal fluid biomarkers (Sembill et al., 2023) as well as identification of a range of clinical courses associated with CAA such as amyloid-related imaging abnormalities (ARIA) during anti-amyloid immunotherapy, spontaneously occurring CAA-related inflammation (CAA-ri) and iatrogenic CAA (iCAA). However, there are still many aspects of the disease that require extensive research. For example, disease modifying treatments, the natural course of the disease in hereditary vs. sporadic forms, screening methods for those at higher risk, modifiable and non-modifiable risk factors, trajectory after treatment, preventive measures and so on should be addressed in future research.

Currently, there is no cure for CAA and its underlying mechanism as well as risk factors are under investigation but not fully elucidated. Therefore, an update on new research findings is timely. As a precursor to the 8th International Cerebral Amyloid Angiopathy Conference, Perth, Western Australia [3–5th November 2022 (Martins et al., 2022)], the aim of this Frontiers Research Topic was to provide an update on CAA research including basic and translational projects into understanding its underlying mechanisms, risk factors, natural history and pathogenesis pathways. Such research is expected to inform screening methods, neuro-pathophysiology, biomarkers, as well as preventive and treatment interventions.

Under this Research Topic, we have several interesting and informative publications. Storti et al. provide a brief review of various features of the CAA-ri and iCAA. In a related but independent paper, Muller describes the case of a 56-year-old female with probable iCAA. Horn et al. examined the peak width of skeletonized mean diffusivity (PSMD) and have reported a solid predictive value for PSMD as compared to other MRI markers. However, in another study in this Research Topic, Chen et al. examined the atrophy of subcortical volumes in CAA, Alzheimer's and healthy control participants and reported non-significant findings for the reduction of subcortical volumes, in contrast to previous studies. They also reported higher PSMD in CAA participants as compared to those with Alzheimer's disease and healthy controls. As a small vessel disease, one would expect CAA does result in higher brain iron content that can be assessed using quantitative susceptibility mapping (QSM) on MRI. However, the study by Sharma et al. published here, did not report such results nor did they find a significant relationship between QSM and cognitive outcome measures. Finally, Savar et al. provided an update on the current state of fluid biomarkers in CAA and the potential future research directions in this area.

In conclusion, while there has been significant increase in CAA research with tremendous findings, as noted in the publications under this Research Topic, we still have many questions and uncertainties about “bench to bedside” of CAA. We are looking forward to upcoming research findings that can inform underlying mechanism and treatment modalities but also screening and identification methods for those who are at higher risk of CAA.

## Author contributions

HS: Writing – original draft, Writing – review & editing. SG: Writing – review & editing. LW: Writing – review & editing.
